# Primary Sclerosing Encapsulating Peritonitis (PSEP) With Meckel’s Diverticulum: A Rare Case Report

**DOI:** 10.7759/cureus.39756

**Published:** 2023-05-30

**Authors:** Vimlendra K Chaudhary, Anubhav Vindal, Vineel Sai Deepak Kallepalli, Vivek Deep, Manu Vats, Gautam Chellani, Mohd. Nayab Ansari

**Affiliations:** 1 Department of Surgery, Maulana Azad Medical College, New Delhi, IND

**Keywords:** meckel´s diverticulum, abdominal pain, small intestine, ascites, intestinal obstruction, abdominal cocoon, sclerosing encapsulating peritonitis

## Abstract

Sclerosing encapsulating peritonitis (SEP) is a rare disease. Preoperative diagnosis of SEP can be made with imaging, such as computed tomography (CT). SEP is characterized by a partial or complete encasement of the small intestine by a layer of a thick grayish-white fibro collagenous membrane similar to an abdominal cocoon. The most common symptoms of SEP are abdominal pain, nausea, and vomiting. This rare disease often leads to acute or sub-acute intestinal obstruction. We discuss, in this report, how we managed a case of primary sclerosing encapsulating peritonitis with Meckel's diverticulum at our institution.

## Introduction

Sclerosing encapsulating peritonitis (SEP) is a rare cause of intestinal obstruction characterized by the small intestine being partially or completely encased by a fibrous membrane [1]. Owtschinnikow [2] first reported this disease as “peritonitis chronica fibrosa incapsulata” in 1907, while Foo et al. first used the name “abdominal cocoon” in 1978 [3]. The prevalence of SEP is not known. Akbulut provided a detailed classification of SEP, where they divided it into primary and secondary forms according to the underlying etiology [4]. Primary SEP (PSEP) has also been termed idiopathic SEP or abdominal cocoon, and their variety has no definite cause. Secondary SEP always has a history of peritoneal dialysis, abdominal tuberculosis, medicine intake, abdominal surgery, or organ transplantation. The highest risk factor for secondary SEP is peritoneal dialysis [5]. Here, we report a rare case of PSEP with a concomitant Meckel’s diverticulum.

## Case presentation

A 15-year-old girl presented to surgical OPD with complaints of multiple episodes of generalized pain abdomen for the last one and a half years. The pain was sudden in onset, colicky in nature, non-radiating, and non-migrating. It was aggravated by the consumption of food. The patient had to induce vomiting to get relief from the pain. She also complained of constipation and weight loss for the last one and a half years. There was no history suggestive of tuberculosis or any other systemic co-morbidities. The patient had attained menarche at the age of 12 years with a regular menstruation cycle.

**Figure 1:**
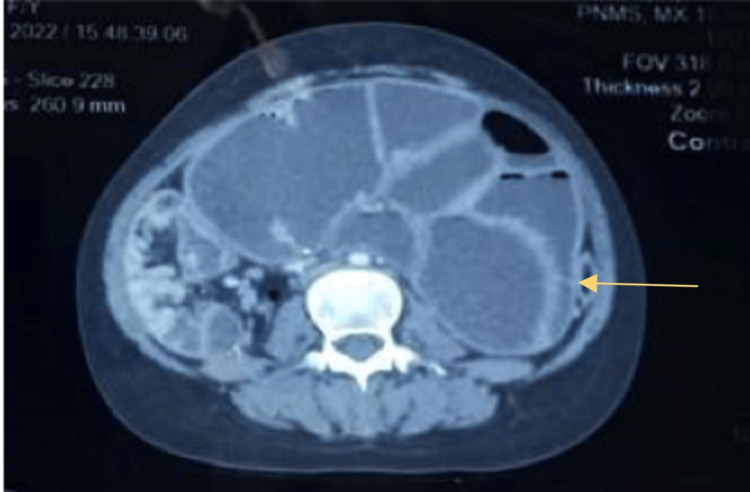
CECT abdomen - axial section CECT - contrast-enhanced computed tomography

**Figure 2:**
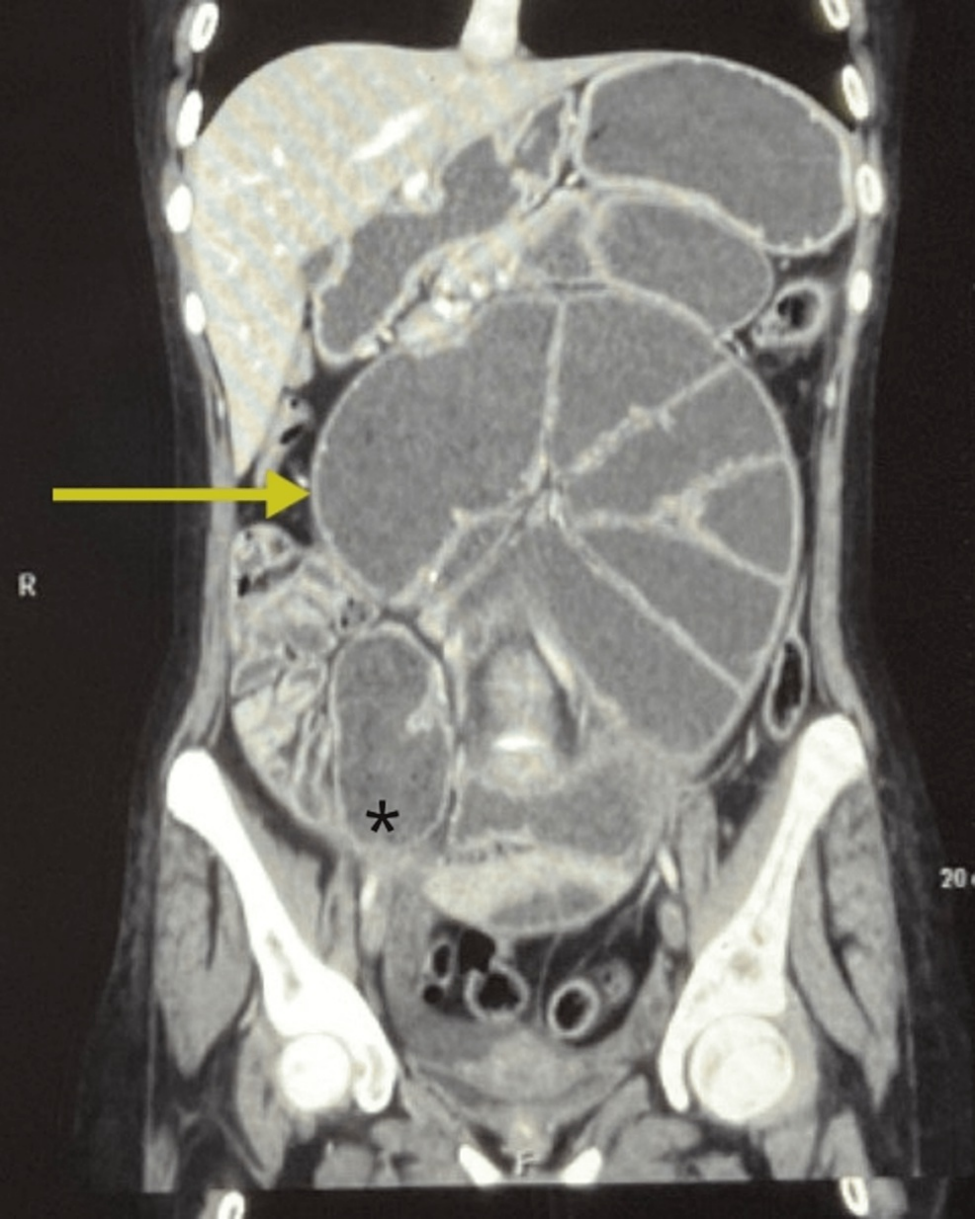
CECT abdomen - coronal section Circumferential homogenously enhancing long segment (single arrow) (4.4 cm) wall thickening (7.3 mm) suggestive of small bowel stricture seen in the proximal ileal loop. A similar appearing stricture is seen in the mid-ileal loops as well (3 cm in length and 7 mm in thickness- double arrow). Jejunal loops are grossly dilated (6.2 cm) with multiple air-fluid levels and are seen in the central abdomen. The stomach and duodenum also appear distended. There is the presence of collapsed ileal loops lateral and posterior to the caecum (marked with *). CECT - contrast-enhanced computed tomography

A general physical examination revealed a pulse rate of 80/minute and blood pressure of 106/78mmHg. There was no cervical or inguinal lymphadenopathy. The abdomen had generalized distension and was non-tender. An ill-defined lump was palpable in the central abdomen. Per rectal examination revealed fecal staining of the gloved finger. The rest of the systemic examination was within normal limits. The patient had got few radiological investigations before presenting to our institution. On reviewing these, the x-ray abdomen showed multiple air-fluid levels with dilated small bowel loops suggestive of intestinal obstruction. A contrast-enhanced computed tomography (CECT) of the whole abdomen was performed which revealed circumferential homogenously enhancing mural thickening of proximal and mid-ileal loops with dilated jejunal loops suggestive of ileal stricture causing small bowel obstruction. It also revealed the presence of collapsed ileal loops lateral and posterior to the caecum along with peri-caecal hernia (Figures 1, 2).

 ​​​​​​

**Figure 3:**
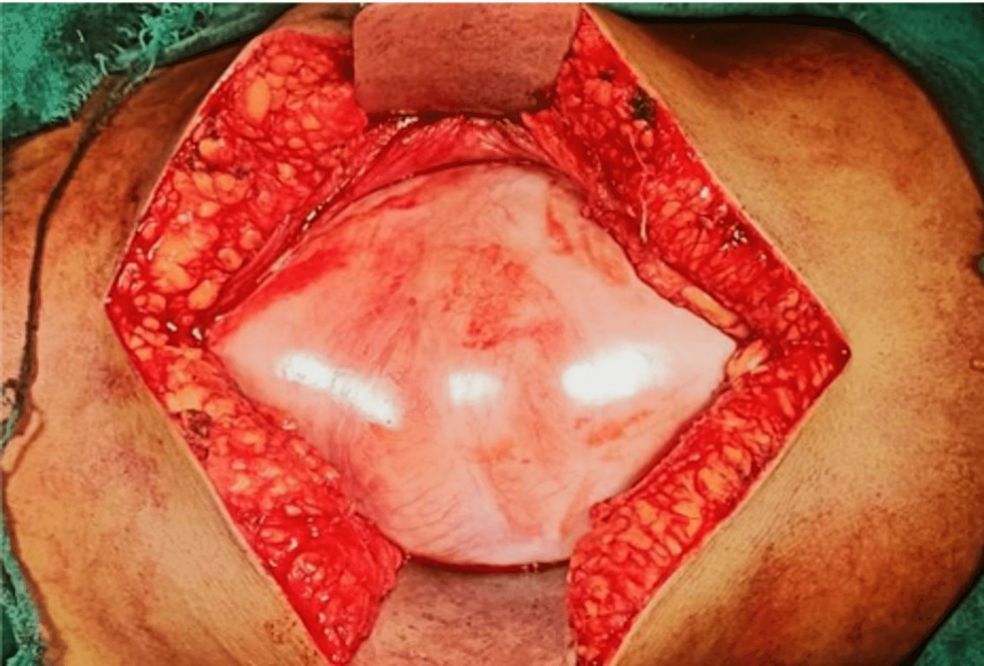
Greyish-white thick membrane covering the small bowel

**Figure 4:**
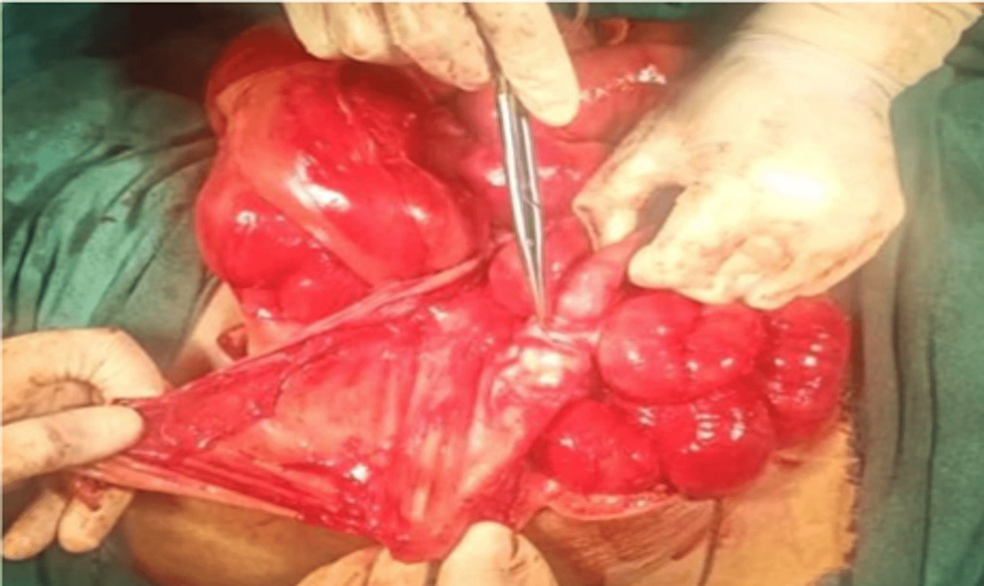
Non-inflamed Meckel's diverticulum found 40 cm proximal to ileocecal junction

After a thorough evaluation of the patient and with a working diagnosis of sub-acute intestinal obstruction due to a jejunal stricture, the patient was taken up for exploratory laparotomy on an elective basis. During exploration, it was found that the whole of the small bowel was covered with a greyish-white thick membrane (Figure 3). There was no free fluid in the abdomen. After a meticulous and painstaking dissection, the small bowel was freed from the membrane. During adhesiolysis, a Meckel’s diverticulum was discovered about 40 cm proximal to the IC junction. Since it had a wide base and was not inflamed, no intervention was done (Figure 4). Ileal loops were found behind the caecum suggestive of paracaecal hernia. The rest of the intra-abdominal findings were essentially normal. Postoperative stay was uneventful.​​​​​​

## Discussion

SEP was first described more than a century ago in the year 1921. SEP was initially termed as peritonitis chronica fibrosa incapsulata to describe the membrane encasing the intestine. It has since also been named “icing sugar” bowel and fibroblastic peritonitis [2]. Several terms are currently used to describe the condition in which a membrane encases the gut, including peritoneal encapsulation (PE), abdominal cocoon, and idiopathic and secondary SEP [4]. The fibro collagenic membrane can encase additional organs such as the large intestine, liver, and stomach leading to acute, subacute, or chronic episodes of intestinal obstruction, loss of appetite, weight loss, fever, and ascites [6,7]. It can be primary (idiopathic) or secondary. Secondary variety is more common and includes encapsulation due to chronic peritoneal dialysis, ventriculoperitoneal shunts, infections such as abdominal tuberculosis, drugs including peritoneal chemotherapy, beta-blockers like practolol, and systemic inflammatory conditions like sarcoidosis to name a few [6].

Idiopathic or PSEP is a condition that affects young adolescent females more commonly in tropical countries [4]. Many theories have been proposed to explain the pathogenesis of PSEP. Retrograde menstruation with superimposed viral infection, retrograde peritonitis, and cell-mediated immunological tissue damage caused by gynecological infection are some of these. However, because this syndrome has been shown to affect men, premenopausal women, and children, there appears to be little support for these hypotheses [7]. Further research is required to elucidate the exact cause in these patients.

The present case is of a young adolescent female who was diagnosed with PSEP with subacute intestinal obstruction. It is difficult to obtain a pre-operative diagnosis due to the non-specific clinical manifestations [8]. In the present case, the diagnosis was established intraoperatively. Investigations that may aid in making a diagnosis include a barium x-ray, which shows a characteristic cauliflower appearance due to clumped ileal loops, and a CT scan which was suggestive of small bowel loops encapsulated by a thick, rigid membrane [9]. But these findings have less sensitivity in the presence of non-specific clinical symptoms, the diagnosis is thus mostly confirmed intra-operatively. In the present case, in addition to PSEP, there was a noninflamed Meckel’s diverticulum along with a paracaecal hernia.

An extensive search of the published literature revealed only one reported case of PSEP with Meckel’s diverticulum. In this pattern, decapsulation and adhesiolysis of all encapsulated segments were performed as the first intra-operative treatment steps, which were followed by the release of the imprisoned intestinal segments and the closure of the hernia sac's mouth. The ileal portion that was conglomerated and perforated was then removed [10].

The histopathological examination of the encapsulating thick membrane removed from this patient showed the presence of fibrous tissue. The mainstay of surgical management is adhesiolysis and partial or complete excision of the peritoneal membrane depending on the extent of the disease. Peritoneal adhesions can be of three types (type I: the membrane encases a portion of the intestine; type II: the entire intestine; and type III: in addition to the intestine other organs are encapsulated by the membrane (appendix, colon, etc). Bowel resection has not yielded promising results [9].

## Conclusions

To conclude, PSEP is a rare condition and a diagnostic challenge. Although cases of both primary and secondary SEP have been reported in the literature, a high index of suspicion by the surgeon is essential for clinching the diagnosis early. Meckel's diverticulum with PSEP is even more rare.
